# Unravelling the Role of *Candida albicans* Prn1 in the Oxidative Stress Response through a Proteomics Approach

**DOI:** 10.3390/antiox13050527

**Published:** 2024-04-26

**Authors:** Victor Arribas, Lucia Monteoliva, María Luisa Hernáez, Concha Gil, Gloria Molero

**Affiliations:** 1 University of Salamanca (USAL), 37008 Salamanca, Spain; varrib01@ucm.es; 2 Department of Microbiology and Parasitology, Faculty of Pharmacy, Complutense University of Madrid (UCM), 28040 Madrid, Spain; luciamon@ucm.es (L.M.); gloriamolero@ucm.es (G.M.); 3 Ramon y Cajal Health Research Institute (IRYCIS), 28034 Madrid, Spain; 4 Proteomics Unit, Biological Techniques Center, Complutense University of Madrid (UCM), 28040 Madrid, Spain; mlhernae@ucm.es

**Keywords:** *C. albicans*, Prn1, oxidative stress response, Pirin, proteomics, apoptosis, proteasome, Mnl1, Nrg1, Cub1, mitochondria

## Abstract

*Candida albicans* Prn1 is a protein with an unknown function similar to mammalian Pirin. It also has orthologues in other pathogenic fungi, but not in *Saccharomyces cerevisiae*. Prn1 highly increases its abundance in response to H_2_O_2_ treatment; thus, to study its involvement in the oxidative stress response, a *C. albicans prn1∆* mutant and the corresponding wild-type strain SN250 have been studied. Under H_2_O_2_ treatment, Prn1 absence led to a higher level of reactive oxygen species (ROS) and a lower survival rate, with a higher percentage of death by apoptosis, confirming its relevant role in oxidative detoxication. The quantitative differential proteomics studies of both strains in the presence and absence of H_2_O_2_ indicated a lower increase in proteins with oxidoreductase activity after the treatment in the *prn1∆* strain, as well as an increase in proteasome-activating proteins, corroborated by in vivo measurements of proteasome activity, with respect to the wild type. In addition, remarkable differences in the abundance of some transcription factors were observed between mutant and wild-type strains, e.g., Mnl1 or Nrg1, an Mnl1 antagonist. orf19.4850, a protein orthologue to *S. cerevisiae* Cub1, has shown its involvement in the response to H_2_O_2_ and in proteasome function when Prn1 is highly expressed in the wild type.

## 1. Introduction

*Candida albicans* is a dimorphic fungus which is part of the commensal human microbiota. However, under certain conditions, this opportunistic pathogen causes systemic infections, especially in immunocompromised patients [1,2]. Invasive candidiasis is one of the main nosocomial diseases, particularly among patients in intensive care units, making it a major public health concern. It is estimated that more than 1.5 million people around the world are affected by invasive *Candida* infection per year, with a resulting mortality of 63.6% [3]. As a result, the World Health Organization (WHO) has included this pathogen in their priority list of fungal pathogens [4,5,6].

During invasive candidiasis, *C. albicans* interacts with phagocytes, mainly macrophages, which produce oxidant molecules such as hydrogen peroxide (H_2_O_2_) and nitric oxide (NO) in response to the infection. Successful *C. albicans* infection depends, to a significant degree, on the resistance to this oxidative attack [7,8,9]. In the end, the action of phagocytes leads 30% of *C. albicans* cells to undergo programmed cell death by apoptosis [9]. Previous studies have shown that *C. albicans* exhibits important genome, transcriptome, and proteome remodeling during the interaction with macrophages [10,11]. These changes include the enrichment of proteins with oxidoreductase and superoxide dismutase activities, which counteract the high ROS levels in the cell. Yeast cells also experience an increase in the metabolism of amino acids and nucleotides, in contrast to a reduction in glycolysis and translation [10]. The high-osmolarity glycerol (HOG) signaling pathway is mainly responsible for these changes in *C. albicans* together with several transcription factors, including Cap1 and Hap43 [12,13,14,15]. To study *C. albicans’s* oxidative response, treatment with H_2_O_2_ provides an excellent strategy to reproduce the oxidative stress induced by phagocytes. This agent has been used in a recent quantitative proteomics analysis from our group, enabling the identification of new proteins likely involved in the detoxification process, such as Prn1 or Oye32 [16].

Prn1 is a protein that shares similarities with mammalian Pirin but with an unknown function in *C. albicans*. Pirin expression correlates with the activation of antioxidant transcription factor Nrf2 and the subsequent expression of related proteins, such as NAD(P)H oxidoreductase 1 (NQO1) [17,18]. Pirin also contains a cupin-activating domain that enables binding to metal ions, such as iron or copper, changing its conformation from the inactive form (Fe^2+^ binding) to the active form (Fe^3+^ binding) [19]. This activation is crucial to modulate the response to different stresses, such as oxidative stress or cell death by apoptosis [20]. A similar protein, PirA, has been identified in a prokaryotic microorganism, *Streptomyces*, as a negative regulator of the mitochondrial beta-oxidation pathway involved in the reduction of oxidative stress [21]. Thus, Prn1 may be related to the oxidative stress response and, in consequence, to virulence. In *C. albicans*, *PRN1* has three homologues, PRN2, PRN3, and PRN4, the latter being the most similar to *PRN1*. On the other hand, while some uncharacterized orthologues are predicted in other *Candida* species, such as *C. dublinensis* or *C. parapsilosis,* and in other fungi, such as *Aspergillus nidulans*, or *Neurospora crassa*, no orthologues are found in *C. auris* or *S. cerevisiae*.

To shed light on the role of Prn1 in the oxidative stress response, we have used a null *C. albicans prn1∆* mutant and the corresponding wild-type strain SN250 [22] to study the effect of H_2_O_2_ in the presence or absence of Prn1. Phenotypic studies and a data-dependent acquisition (DDA) quantitative proteomics approach are described in the present work. We have shown that Prn1 absence induces an increase in ROS levels and an increased level of apoptosis and cellular death. To compensate Prn1 absence, *C. albicans* increases other molecular mechanisms, for instance, proteasome activity. In addition, transcription factors such as Mnl1 exhibit differential behavior between the wild type and the deleted mutant strain. Prn1, as deduced from our results, is important for *C. albicans’s* oxidative stress response. Proteomic studies have allowed the identification of the previously undescribed *C. albicans* protein corresponding to orf19.4850, a gene orthologue to *S. cerevisiae CUB1*, the protein of which had increased abundance only in the wild type, where Prn1 is expressed under oxidative stress.

## 2. Materials and Methods

### 2.1. Fungal Strains and Culture Conditions

A *Candida albicans* null *prn1∆* mutant and the corresponding wild-type strain (SN250) from Noble’s collection [22] were used for Prn1 function characterization assays. *C. albicans* wild-type SC5314 and *prn1∆*/*PRN1* and orf19.4850∆/orf19.4850 mutant strains from Deming Xu’s collection were used for orf19.4850 function characterization assays. Cells were grown in a conventional YPD-rich medium (1% yeast extract, 2% peptone, and 2% glucose) at 30 °C until reaching the exponential phase (OD600 = 0.8–1). Later, H_2_O_2_ was added at the appropriate times and concentrations, being incubated again at 30 °C during rotatory shaking (180 rpm). H_2_O_2_ (ITS, Sigma-Aldrich, St. Louis, MO, USA) concentrations ranged from 5 to 7 mM for cell viability assays to 80 mM in the drop growth assay. For growth curves, 10 µL of *C. albicans* culture (OD600 = 0.04) were added to 180 µL of the YPD medium and incubated for 72 h at 30 °C with shaking. Regarding the drop growth assays, cells were treated with 80 mM of H_2_O_2_ and 4 µL of yeast culture were spotted every 3 min in YPD agar plates and incubated for 24 h at 30 °C.

### 2.2. Experimental Design

A total of 16 samples from 4 biological replicates of both strains (wild-type and *prn1∆*) in each condition (control or treated) were obtained to perform the proteomics assay. *C. albicans* SN250 and *prn1∆* strains were incubated for 200 min with 10 mM of H_2_O_2_. Analysis for significant protein abundance changes between treated and respective control samples were performed through a statistical nested *t*-test applying q-values < 0.05. Determinations of significant protein abundance changes between both treated samples were also performed through a statistical *t*-test applying q-values < 0.05.

### 2.3. Cell Disruption and Protein Extract Quantification

Control and treated cells were harvested and washed thrice in PBS. Next, lysis buffer (50 mM Tris-HCl [pH 7.5], 1 mM EDTA, 1 mM DTT, 150 mM NaCl, 10% protease inhibitors (Thermo Scientific, Waltham, MA, USA), and 5 mM phenylmethylsulfonyl fluoride [PMSF]) was added to resuspend cell pellet. For the proteasome activity assay, no protease inhibitors or PMSF were added. Cell extracts were obtained by mechanical shaking in a Fast-Prep system (Bio101, Savant, Thermo Fisher, Waltham, MA, USA) over 5 cycles of 30 s, applying glass beads (0.5 to 0.75 mm diameter). The samples were centrifuged for 15 min at 13,000 rpm to separate protein extracts from the cell debris, and protein concentrations were measured using a Bradford assay.

### 2.4. Proteomics Assay

After cell disruption, peptide digestion was performed using 50 μg of protein extracts (iST kit, PREOMICS, Planegg, Germany) [23]. In brief, samples were denaturalized, reduced, and alkylated; later, they were digested, applying a trypsin/LysC mix, and peptides were purified using a reversed-phase LC-MS column. The final peptide concentration of the samples was quantified by fluorimetry using a Qubit4 system (Thermo Scientific, Waltham, MA, USA). They were dried via vacuum centrifugation (SpeedVac, Savant, Thermo Scientific, Waltham, MA, USA) and were reconstituted to a concentration of 0.2 µg/µL of 2% ACN and 0.1% formic acid, and they were stored at −20 °C until analysis. The peptides (1 μg) were analyzed via liquid nanochromatography (nano Easy-nLC 1000, Thermo Scientific, Waltham, MA, USA) coupled to a Q-Exactive HF high-resolution mass spectrometer (Thermo Scientific, Waltham, MA, USA). The peptides were concentrated on-line via reversed-phase chromatography using an Acclaim PepMap 100 guard column (Thermo Scientific, Waltham, MA, USA) and were then separated on a Picofrit C18 reversed-phase analytical column (Thermo Scientific, Waltham, MA, USA). MS/MS data were acquired in the data-dependent acquisition (DDA) mode of the MS. Mass spectra were acquired in a Q-Exactive HF hybrid quadrupole-Orbitrap mass spectrometer (Thermo Scientific, Waltham, MA, USA) for full-MS data-dependent acquisition (DDA) in positive mode with Xcalibur 4.5 software. MS scans were acquired at an *m*/*z* range of 350 to 1800 Da followed by a data-dependent MS/MS scan (with a threshold of 0.01) of the 15 most abundance precursors with charges of 2–5 in MS scans for high-energy collision dissociation (HCD) fragmentation, with a dynamic exclusion of 10 s and a normalized collision energy (NCE) of 20. Peptide spectrum matches were filtered to a false-discovery rate (FDR) of >1%. Mass spectra (raw files) were processed using Proteome Discoverer v2.4 software (Thermo Scientific Waltham, MA, USA) with the MASCOT v.2.8 search engine, using the *Candida* Genome Database (release 2020_06, 6209 sequences) (CGD) [24] for protein identification to generate a sample-specific peptide list. Mascot version 2.6 was used for the characterization and quantitative analysis of the *Candida* peptides. The search parameters included the carbamidomethylation of cysteines as a fixed modification; the oxidation of methionine and N-terminal acetylation as variable modifications; trypsin as the enzyme; and a maximum of 2 missed cleavages allowed. The precursor mass tolerance was 10 ppm and the fragment mass tolerance was 0.02 Da. The validation was based on q-values from the Percolator algorithm, with an FDR > 0.01.

### 2.5. Protein Quantification

To determine the abundance of the identified peptides and proteins in different isolates, a label-free experiment based on precursor signal intensity was performed. The processing workflow was initiated with the recalibration of masses through a rapid search in Sequest HT (Thermo Scientific, Waltham, MA, USA) against the database and based on the positive identifications; the chromatograms of all the samples were aligned with a tolerance of up to 10 min. Subsequently, the alignment of the retention times between the different samples analyzed for the quantification of the precursor ions was performed, taking into account the unique peptides present in at least 50% of the replicates. Finally, the results were normalized to the total amount of the peptides, equaling the total abundance among the different samples.

### 2.6. GO Enrichment Analysis and Protein Clustering

GO enrichment analysis was carried out using the GO Term Finder and GO Slim Mapper tools from the *Candida* Genome Database (CGD) [25] according to the biological process, molecular function, and cellular component. A protein network cluster analysis was performed using STRING v.12.0 software.

### 2.7. Viability Assays

*C. albicans* cells were harvested after 200 min in the presence of 10 mM of H_2_O_2_, and then 5 µL of propidium iodide (PI) (50 µg/mL) was added for 5 min at room temperature. The percentage of cell death was measured by flow cytometry and the results were analyzed using the FlowJo v.10.9 software. Statistical analysis was performed using a *t*-test.

### 2.8. ROS Detection

*C. albicans* cells were incubated for 200 min with 10 mM of H_2_O_2_ and then washed thrice with cold PBS. Intracellular ROS were detected by staining treated cells with dihydrorhodamine 123 (DHR-123) (ITS, Sigma-Aldrich, St. Louis, MA, USA) at a 5 μg/mL final concentration for 30 min. ROS were detected by fluorescence microscopy, counting > 200 cells in each of the 3 biological replicates. Fiji—ImageJ v.2.14 software was utilized to quantify fluorescence signals, taking into account the cell volume and subtracting the background fluorescence of the image. Statistical analysis was performed using a *t*-test.

### 2.9. Cellular Apoptosis

SN250 and *prn1∆* strains were cultured and treated with 10 mM of H_2_O_2_ for 50 min. Phosphatidylserine (PS) externalization was measured using Annexin V-fluorescein isothiocyanate (FICT) in the protoplast cells obtained, applying standard techniques. Briefly, *C. albicans* cells were resuspended in a buffer containing 50 mM of K_2_HPO_4_, 50 mM of dithiothreitol (DTT), and 5 mM of EDTA (pH 7.2) at 30 °C. Then, a buffer composed of 50 mM of KH_2_PO_4_, 40 mM of 2-mercaptoethanol, 0.15 mg/mL of zymolyase 20T, 20 mL of glusulase, and 2.4 M of sorbitol was added and incubated for 30 min. Protoplasts were stained following ApoAlert kit indications (TaKaRa Bio, Ann Arbor, MI, USA), and the ratio of apoptotic cells was measured by flow cytometry. The results were analyzed using the FlowJo software. Statistical analysis was performed using a *t*-test (*p*-value < 0.05).

### 2.10. Mitochondrial Function

Mitochondrial membrane depolarization was measured before and after the treatment of *C. albicans* SN250 and *prn1∆* cells with 10 mM of H_2_O_2_ for 15, 25, or 50 min. The cells were resuspended in PBS and then JC-1 dye was added at a final concentration of 0.25 µL/mL and incubated for 20 min. The results were analyzed by flow cytometry using the FlowJo software.

### 2.11. Proteasome Activity

SN250 and *prn1∆* cells were treated with 10 mM of H_2_O_2_ for 200 min. Total extracts were obtained as previously described, and later, proteasome chymotrypsin-like protease activity was measured in a fluorometric assay using Proteasome LLVY-R110 substrate (Proteasome 20S activity assay kit; Sigma-Aldrich). The substrate was added to 100 µg of total extracts for 2 h at 30 °C, according to the manufacturer recommendations. Fluorescence signals were measured with BMG FLUOstar Galaxy (BMG Labtech, Ortenberg, Germany) (λex = 480 to 500 nm/λem = 520 to 530 nm). Statistical analysis was performed using a Mann–Whitney test.

## 3. Results

### 3.1. Impact of PRN1 Deletion on Cell Death and Recovery under Oxidative Stress

**Figure 1:**
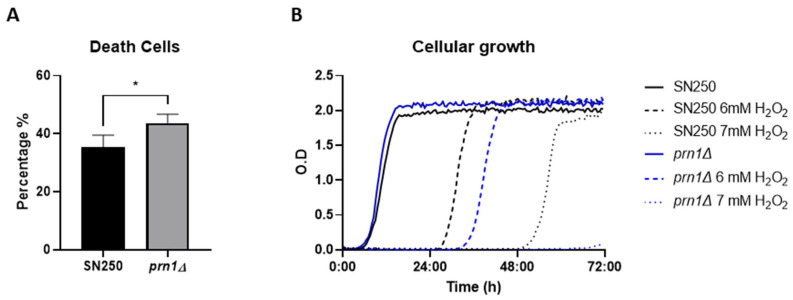
*C. albicans* SN250 and *prn1∆* cell death and growth in the presence of H_2_O_2_. (**A**) Percentage of propidium iodide (PI)-positive dead cells measured by flow cytometry after 200 min in the presence of 10 mM of H_2_O_2_. Results represent the average of three biological replicates. Error bars indicate standard deviation. * *p* < 0.05, unpaired *t*-test. (**B**) Growth curves of both strains in the presence of 6 mM and 7 mM of H_2_O_2_. The graph presents the most representative curve of three biological replicates.

Cell death during oxidative stress was quantified by propidium iodide (PI) staining after 200 min in the presence of 10 mM of H_2_O_2_ for the *prn1∆* and SN250 strains. Flow cytometric analyses showed that up to 45% of cells in the *prn1∆* strain were dead after treatment compared with 35% for the SN250 strain (Figure 1A). This increased percentage of dead cells correlates with the delay in cell growth of nascent cultures of both strains after exposure to H_2_O_2_ in a dose-dependent manner. This delay was more pronounced for the *prn1∆* strain (Figure 1B). These experiments show the importance of Prn1 for the viability and recovery of *C. albicans* after H_2_O_2_ treatment.

### 3.2. Proteomic Response of SN250 and prn1∆ Strains to H_2_O_2_ Treatment

**Figure 2:**
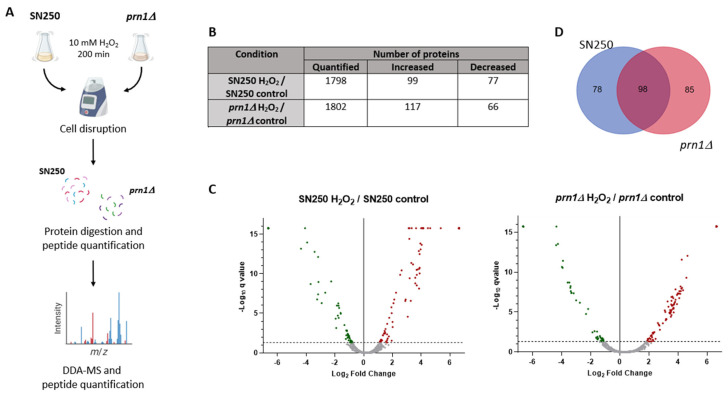
Quantitative proteomics assay (DDA-MS) of SN250 and *prn1∆* strains in response to 200 min of 10 mM H_2_O_2_ treatment. (**A**) Workflow of quantitative proteomics assay (DDA-MS) to compare SN250 and *prn1∆* strains after 200 min 10 mM H_2_O_2_ treatment. After cell disruption, protein cell extracts of each strain are digested and quantified; later, peptide samples are analyzed in the mass spectrometer. Images were created with BioRender. (**B**) Number of quantified proteins and proteins showing significant differences in abundance between the treated and non-treated (control) conditions for each strain. (**C**) Volcano plots representing proteins with significant changes in abundance. Significant changes in the protein abundance (−log10 q-value > 1.3) after treatment are presented in red for increase or green for decrease. (**D**) Venn diagram showing common and non-common proteins with significant changes in abundance between strains in response to the treatment.

To unmask the role of Prn1 in the oxidative stress response, we performed a quantitative proteomics assay (DDA-MS) of the SN250 and *prn1∆* strains after 200 min of H_2_O_2_ treatment and compared it to the control condition (Figure 2A). The proteomics analyses enabled the quantification of approximately 1800 *C. albicans* proteins in the SN250 and *prn1∆* strains using the *Candida* Genome Database (CGD) (Table S1). Statistical analysis of the relative quantification (q-value < 0.05) revealed that 176 and 183 proteins significantly changed their abundance in response to the treatment of SN250 and *prn1∆*, respectively (Figure 2B and Table S2). The volcano plots in Figure 2C represent the different changes in protein abundance between the two strains. As shown in the Venn diagram (Figure 2D), only 98 proteins were common to both comparisons, highlighting the importance of Prn1 in the oxidative response (Table S3).

An analysis of the 10 proteins with the greatest increase in relative abundance for each strain revealed only three proteins in common: Cip1, Ach1, and Pim1 (Table 1). In the SN250 strain, we observed previously described proteins (e.g., Prn1 or Oye22) [16], as well as other proteins that did not significantly increase in the *prn1∆* strain (e.g., Psa2 and Alt1). For the *prn1∆* strain, we detected Qcr9 and Nuc2 proteins implicated in ubiquinone/ubiquinol redox. Qcr9 did not significantly increase in abundance in the wild-type strain, as also occurred with orf19.7310. Particularly interesting was orf19.6035, the abundance of which significantly increased in both strains, but its function remains unknown.

**Figure 3:**
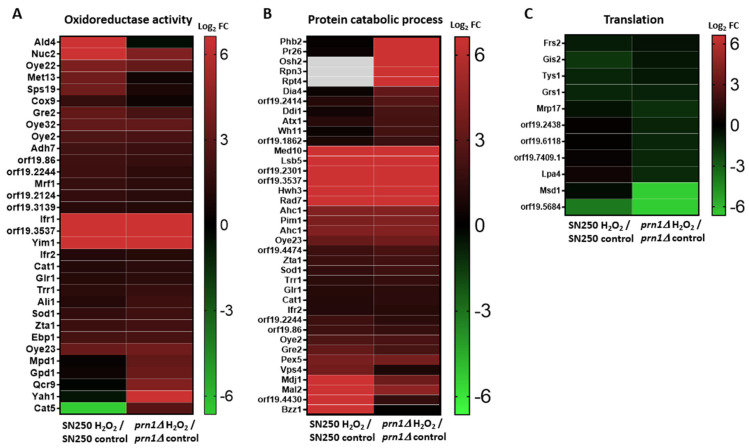
Heat maps of proteins that changed in abundance after 200 min of 10 mM H_2_O_2_ treatment in each strain with respect to the control condition grouped by GO term. (**A**) Oxidoreductase, (**B**) protein catabolic process, and (**C**) translation. Gray gaps indicate proteins not detected in that strain.

Gene Ontology (GO) enrichment for the biological process and function of all proteins with significantly increased abundance in each strain (Table S2) showed a high increase in proteins with the oxidoreductase activity GO term for both strains (Figure 3A), although in a higher number for SN250. Many of these proteins were different for each strain (Table S4). We also observed enrichment of protein catabolic process GO term proteins for both strains in the presence of H_2_O_2_ (Figure 3B). Increased proteins in the *prn1∆* strain were mainly related to the regulatory subunit of the proteasome (Phb2, Pr26, Rpn3, and Rpt4), whereas these proteins in the SN250 strain were chaperones and ubiquitin-binding proteins involved in protein transport from Golgi to vacuole (Bzz1, orf19.4430, Vps4, and Mdj1). The *prn1∆* strain also presented a greater decrease in the abundance of proteins associated with translation than the treated SN250 strain (Figure 3C). Both strains presented a decrease in nucleotide metabolic process GO term proteins, which are related to purine and pyrimidine biosynthesis, and this effect was higher in the *prn1∆* strain (Figure S1). To emphasize the differential response of each strain to oxidative stress, GO term analysis was carried out for the proteins that exclusively increased in abundance for each strain (Figure S2). The 78 proteins that significantly increased in abundance only in the SN250 strain were significantly enriched in oxidoreductase activity proteins, suggesting a higher oxidative stress response when Prn1 is present. On the other hand, GO term enrichment of the 85 proteins that significantly increased in abundance only in the *prn1∆* strain was related to proteasome-activating activity, which could indicate higher proteasome activity (Figure S2).

**Figure 4:**
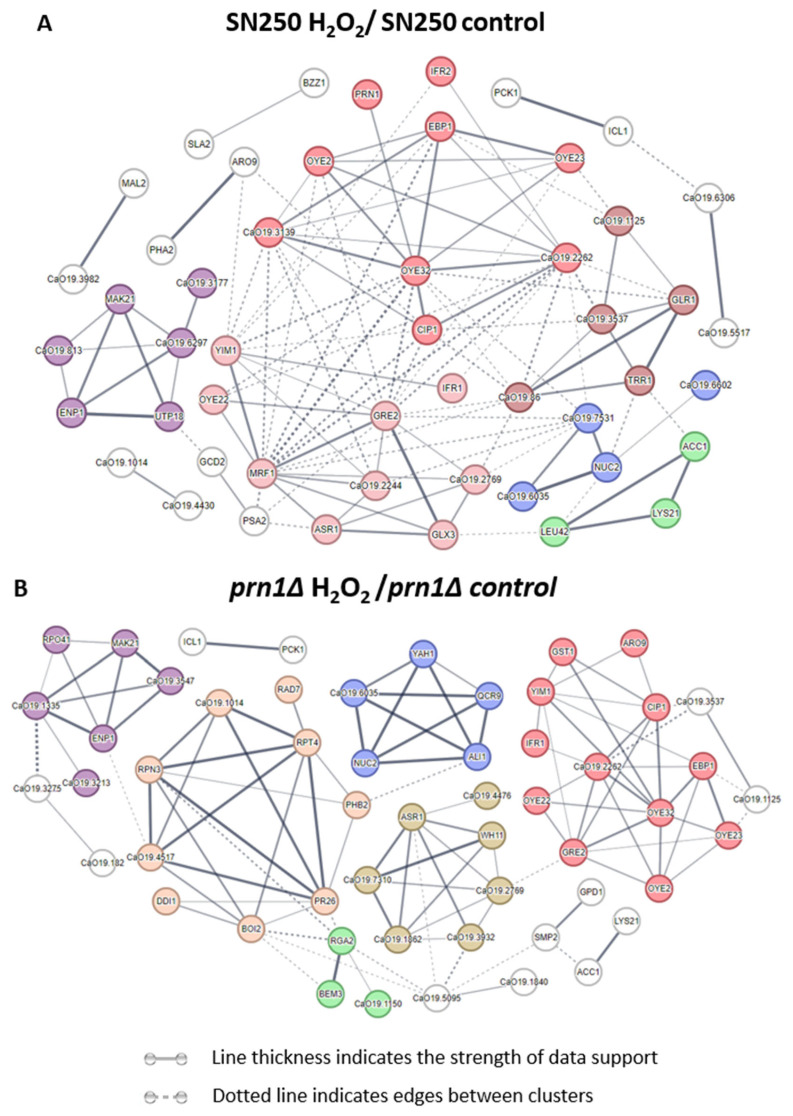
Predicted networks of proteins with increased abundance after H_2_O_2_ treatment in the SN250 and *prn1∆* strains using STRING software. Line thickness indicates the strength of supporting data. Dotted lines indicate edges between clusters. (**A**) Clusters: oxidoreductase function (red, dark red, and light red), pre-ribosome and ribosome biogenesis (purple), dehydrogenase complex of the respirasome (blue), and amino acid biosynthesis (green). (**B**) Clusters: oxidative stress response (red), proteasome regulatory subcomplex (orange), heat shock response (gold), ribosome biosynthesis (purple), mitochondrial oxidoreductase complex (blue), and Rho GTPase regulation (green).

The proteins that significantly increased in abundance in the SN250 and *prn1∆* strains under oxidative treatment (Table S2) were grouped into 13 and 11 predicted protein network clusters, respectively, using the STRING software. In the SN250 strain, we found three clusters related to oxidoreductase function. Other clusters were implicated in pre-ribosome and ribosome biogenesis, the dehydrogenase complex of the respirasome, and amino acid biosynthesis (Figure 4A). For the *prn1∆* strain, we also found protein clusters involved in the oxidative stress response and ribosome biosynthesis, but also in the proteasome regulatory subcomplex, heat shock response, mitochondrial oxidoreductase complex, and Rho GTPase regulation (Figure 4B).

Proteins only detected in one condition but not in the other for each strain are presented in Table S5. Among them, we selected those that did not show any significant change or were not detected in the other strain (Table 2). In the case of the SN250 strain, we highlight riboflavin and carnitine antioxidant biosynthesis-related proteins (Rib2 and Ald4). Interestingly, orf19.4850, a gene orthologue to *S. cerevisiae CUB1* linked to DNA repair and proteasome function [26], encodes a protein that was only detected in the wild-type strain with H_2_O_2_ treatment. For the *prn1∆* strain, we highlight proteins related to catabolism (Osh2, Phb2, Pr26, Rpn3, and Rpt4) and two transcription factors, Tif33 and orf19.1150, that were only detected under oxidative stress. Conversely, among the proteins that were not detected after oxidative treatment in the *prn1∆* strain, we found proteins related to ribosome biogenesis (Afg2, Has1, Lig1, and Ubp12) and the transcription factor/repressor Nrg1.

The most dramatic differences in protein abundance from Table S5 are those with an opposite change between both strains after H_2_O_2_ treatment (Table 3). Among these proteins, we observed two transcription factors (Mnl1 and Bas1), an Mfg1 biofilm growth regulator, and two ubiquinone biosynthesis-related proteins (Cat5 and Fmp53).

### 3.3. Comparative Proteomics Analysis between SN250 and prn1∆ Strains Either in the Presence or the Absence of Oxidative Stress

**Figure 5:**
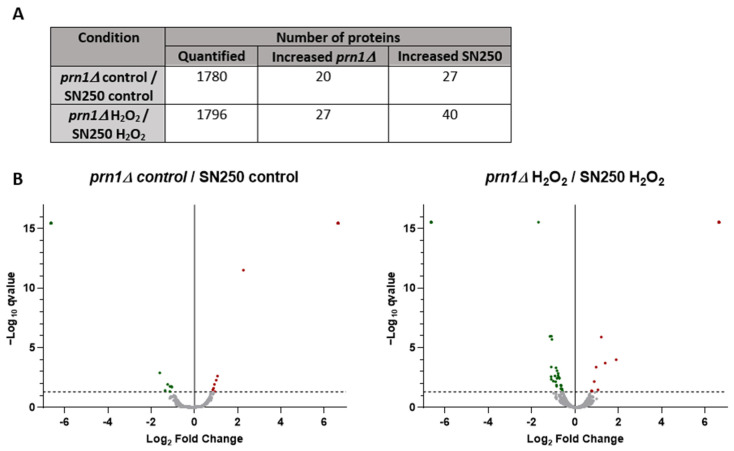
Comparative proteomics analysis between the SN250 and *prn1∆* strains in the control condition and after 200 min of 10 mM H_2_O_2_ treatment. (**A**) Number of quantified proteins and those with differences in abundance for each strain. (**B**) Volcano plots representing proteins with a significantly different abundance between the two strains in each condition. Significant changes in the protein abundance (−log10 q-value > 1.3) are presented in red for *prn1Δ* strain or green for wild-type SN250 strain.

The comparative analysis of the quantified proteins between strains either in the absence or the presence of oxidative stress (Table S1) highlights the possible role of Prn1 under both circumstances. Statistical analysis (q-value < 0.05) uncovered only 47 proteins with a significant change in abundance between both strains without an oxidative agent and 67 proteins after treatment with H_2_O_2_ (Figure 5A and Table S6). Volcano plots pointed out the different changes in abundance after H_2_O_2_ treatment compared with the untreated condition (Figure 5B), supporting an important role of Prn1 in response to this stress.

In the control condition, the mitochondrial GO term proteins with significant changes in abundance between both strains were observed (Figure S3A); in the *prn1∆* strain, they were related to mitochondrial ribosomes (Img1 and orf19.2214) and mitochondrial chaperones (Mdj1 and Hsp78), whereas in the SN250 strain, they were related to protein import into mitochondria (Fmp28 and Tom6), mitochondrial oxidoreductase/cell redox (Phb2 and Yah1), and mitochondrial respiratory chain assembly (orf19.1336.2). This analysis suggests an important role of Prn1 in mitochondrial ROS detoxication under basal conditions. However, no significant changes in mitochondrial membrane potential using JC-1 dye were observed between SN250 and *prn1∆* untreated strains after 15, 25, or 50 min 10 mM H_2_O_2_ treatment (Figure S3B). In addition, two transcription factors (Mnl1 and orf19.1150) and Swi3, a protein involved in chromatin remodeling, exhibited significant changes.

Under oxidative stress, GO term enrichment analysis of the proteins with a significant change in abundance between both strains indicated an increase in proteasome-activating proteins for the *prn1∆* strain and in translation for the SN250 strain, confirming the previously described results.

### 3.4. Influence of Prn1 Deletion on ROS Production and Proteasome Function

**Figure 6:**
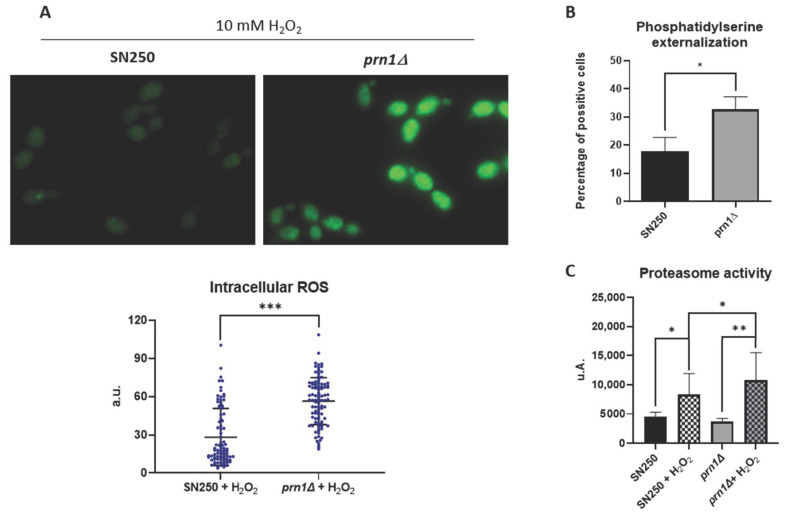
Effect of Prn1 deletion on cell redox homeostasis, apoptosis, and proteasome activity. (**A**) Intracellular ROS levels in the SN250 and *prn1∆* strains after 200 min of 10 mM H_2_O_2_ treatment using the dihydrorhodamine 123 (DHR123) probe. (**B**) Phosphatidylserine (PS) externalization levels after 50 min of 10 mM H_2_O_2_ treatment using Annexin V staining. (**C**) Chymotrypsin-like proteasome activity after 200 min of 10 mM H_2_O_2_ treatment. Results represent the averages of three biological replicates. Error bars indicate standard deviation. * *p* < 0.05, ** *p* < 0.01, and *** *p* < 0.001, unpaired *t*-test.

The GO term enrichment analysis of proteins that vary in abundance after H_2_O_2_ treatment when Prn1 is present suggested that this protein may play an important role in regulating the detoxification process. To functionally validate this result, we measured intracellular ROS levels using a dihydrorhodamine 123 (DHR123) probe after 200 min of 10 mM H_2_O_2_ treatment. The ROS levels were significantly higher in the Prn1-null mutant relative to the wild-type strain (Figure 6A). Oxidative stress-mediated ROS accumulation induces apoptotic cell death [16]. To evaluate the differences between strains, we looked for the first signs of apoptosis, both in the *prn1∆* and in the SN250 strains treated with 10 mM of H_2_O_2_ for only 50 min. Phosphatidylserine (PS) externalization, a marker of apoptosis, was measured by flow cytometry. Annexin V staining showed that 34% of treated *prn1∆* cells presented PS externalization, in contrast to 18% of treated SN250 cells (Figure 6B).

In addition, as previously described, GO term enrichment showed an increase in proteasome-activating proteins in the *prn1∆* strain. Therefore, we examined the activity of the proteasome complex by measuring the chymotrypsin-like protease activity after 200 min of 10 mM H_2_O_2_ treatment. Both strains significantly increased the proteasome activity due to the oxidative treatment, with a significantly higher increase for the *prn1∆* strain (Figure 6C) in concordance with the results of the proteomics assay.

### 3.5. C. albicans orf19.4850 Mutant Response to Oxidative Stress

We detected a protein corresponding to the uncharacterized *C. albicans* orf19.4850, a gene orthologue to *S. cerevisiae CUB1*, exclusively in the SN250 strain under oxidative stress. This suggests that its expression could be related to both oxidative stress and the presence of Prn1.

**Figure 7:**
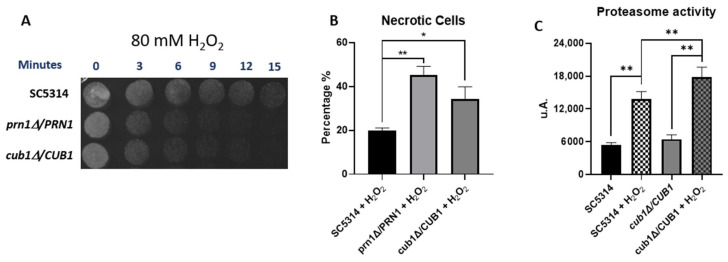
Role of the *C. albicans* protein orthologue to *S. cerevisiae* Cub1 in the oxidative stress response after H_2_O_2_ exposure. (**A**) Drop growth assay of *C. albicans* SC5314, *prn1∆*/*PRN1*, and *cub1∆*/*CUB1* strains treated with 80 mM of H_2_O_2_ for different time intervals. (**B**) Percentage of PI-positive death cells measured by flow cytometry after 200 min in the presence of 10 mM of H_2_O_2_. (**C**) Chymotrypsin-like proteasome activity after 200 min 10 mM H_2_O_2_ treatment. Results represent the averages of three biological replicates. Error bars indicate standard deviation * *p* < 0.05 and ** *p* < 0.01, unpaired *t*-test.

To find out this connection, we measured the susceptibility of the *prn1∆*/*PRN1* and *orf19.4850∆*/*orf19.4850* mutant strains from Deming Xu’s collection and the corresponding wild-type *C. albicans* SC5314 to 80 mM of H_2_O_2_ in a drop growth assay on YPD medium. The growth of both mutant strains was notably reduced (Figure 7A), and flow cytometric analyses after 200 min of 10 mM H_2_O_2_ treatment showed that the percentage of dead cells significantly increased for both mutant strains with respect to the wild-type strain (Figure 7B). In addition, the proteasome activity of the *orf19.4850∆*/*orf19.4850* strain after 200 min of 10 mM H_2_O_2_ treatment also increased significantly with respect to its wild-type control strain (Figure 7C). Thus, the *orf19.4850∆*/*orf19.4850* strain showed similar behavior to the *prn1∆* mutant strain under oxidative stress.

## 4. Discussion

### 4.1. Prn1 Has a Main Role in the Oxidative Response

In a previous quantitative proteomics study on *C. albicans* treated with H_2_O_2_, a high increase in abundance of Prn1 after H_2_O_2_ treatment was described [16]. The function of Prn1 in *C. albicans* remains unknown, but the protein presents partial homology with human Pirin (Figure S5), suggesting a possible conserved function. In this study, the *C. albicans prn1∆* strain showed an increase in dead cells after H_2_O_2_ treatment (Figure 1A) and a delay in cell growth in response to this agent (Figure 1B). All these experiments confirm the importance of Prn1 for cell survival and recovery during oxidative stress, such as mammalian Pirin [27] or *Streptomyces* PirA [21]. Mammal Pirin is functionally similar to the oxidoreductase protein quercetin 2,3-dioxygenase; thus, this protein has been proposed to have intrinsic oxidoreductase enzymatic activity [28]. In-depth *C. albicans* Prn1 sequence analysis using Pfam v36.0 software identified two conserved Pirin domains (Figure S5), which could indicate the same activity.

### 4.2. Prn1 Absence Increases ROS Levels after H_2_O_2_ Treatment

The DDA-MS proteomics approach used in this work showed clear differences in the response of mutant and wild-type strains to oxidative stress (Figure 2 and Table 1). The GO term enrichment (Figure 3 and Table S4) and STRING analyses (Figure 4) of the proteins that significantly increased in abundance for the two strains suggest a stronger oxidative stress response in the wild-type strain. Our hypothesis was functionally validated by the detection of higher intracellular ROS levels for the *prn1∆* strain after H_2_O_2_ treatment (Figure 6A) and a concomitant increase in the percentage of apoptotic cells compared with the wild-type strain (Figure 6B). Concordantly, mammal Pirin has also been related to apoptosis/programmed cell death [29]. The STRING software also detected clusters of mitochondrial oxidoreductase complex and heat shock proteins in the *prn1∆* strain, which suggests that these proteins increase their abundance to counteract the absence of Prn1.

Proteins detected exclusively in the SN250 strain after H_2_O_2_ treatment with an opposite or non-significant change in the *prn1∆* strain (Table 2 and Table 3) likely correlate with the presence of Prn1. A promising protein is Ald4 dehydrogenase, which is necessary for carnitine biosynthesis and is proposed to be an important antioxidant in *S. cerevisiae* [30,31]. Another interesting protein was Rib2, which has pseudouridine synthase activity implicated in riboflavin biosynthesis, a compound with important antioxidant activity in both mammals [32] and yeasts [33]. Ald4 and Rib2 increased in abundance in the SN250 strain after H_2_O_2_ treatment, suggesting that these antioxidants might have been trying to detoxicate the cell. As previously said, Pirin has been proposed to have intrinsic oxidoreductase enzymatic activity [28,34]. Prn1 may also have conserved intrinsic antioxidant activity through riboflavin or carnitine flavonoids, how mammalian Pirin acts on quercetin flavonoids.

In mammalian cells, the active conformation of Pirin (Fe^3+^) is crucial for NF-κB p65 transcription factor binding, which suggests that this protein may connect the oxidative stress response to the proteomic changes associated with this transcription factor [19,20,35]. This could hint at the possible role of Prn1 as a translational coregulator involved in oxidative stress responses or apoptosis in *C. albicans*. A possible relationship between NF-κB and the yeast retrograde response gene (RTG) signaling pathway has been proposed for *S. cerevisiae* [36]. However, we did not identify any significant change in the abundance of RTG pathway components in the wild-type strain in which Prn1 is expressed (Table S7).

Surprisingly, the Prn1 homologues have not shown a redundant function or expression. The proteomics analysis only detected a slight but significant increase in Prn4 abundance (*p*-value < 0.05), which is the most similar to Prn1, in the wild-type strain after H_2_O_2_ treatment, but not in the *prn1∆* strain after treatment, whereas Prn2 and Prn3 were not detected in any of the four conditions. Further studies will be necessary to discover their functions.

### 4.3. Absence of Prn1 Increases Proteasome Activity and Decreases Proteins Related to Translation after H_2_O_2_ Treatment

Enrichment of protein catabolic process GO term proteins was observed in both strains, probably due to the increase in free ROS after H_2_O_2_ treatment, a fact previously described for the *C. albicans* SC5314 strain [16]. In the SN250 strain, these proteins were related to the transport of proteins from Golgi to vacuole within the ubiquitin-dependent protein catabolic processes, while for the *prn1∆* strain, they were related to proteasome regulatory subunits (Pr26, Rpt4, and Rpn3). Some of these proteins were only detected in the treated *prn1∆* strain or only significantly increased in the mutant (Figure 3B and Table 2, Table 3, and Table S2). The higher proteasome activity in the mutant strain was functionally validated (Figure 6C), suggesting that it is necessary to counteract the loss of Prn1’s antioxidant function.

A decrease in *C. albicans* proteins related to translation has been observed during the interaction with macrophages [9,11]. In our experiment, we observed a clear decrease in the abundance of proteins related to both the translation GO term and the nucleotide metabolic process GO term in the *prn1∆* strain, even in basal conditions, but this was not so accentuated in the SN250 strain (Figures S1 and S3B). The higher ribosome biosynthesis and protein translation in the wild-type strain, which was probably related to faster cell recovery after stress, suggest that Prn1 could also be important in the response to host phagocytes.

### 4.4. Differential Expression of Transcription Factors between Strains

Proteins detected only in one condition for each strain (Table 2 and Table 3) revealed drastic differences in the abundance of transcription factors (Mnl1, Nrg1, Bas1, Tif33, and orf19.1150) not described in a recent work on *C. albicans* [37]. The mechanisms regulated by Tif33 or orf19.1150 have not been completely described. Bas1, which is implicated in filamentous growth and virulence, presented an inverse change in abundance between both strains, increasing in the *prn1Δ* mutant but not being detected in the wild-type strain in response to H_2_O_2_. The same pattern of expression was found for Mfg1, a regulator of filamentous growth involved in virulence. Mnl1 is involved in weak acid stress responses, regulating the expression of several genes, including Prn1 [38]. Increased *PRN1* transcription was detected in *C. albicans* biofilms in an acidic environment (pH 4) [39].

Mnl1 was only detected in the *prn1∆* strain under oxidative stress, where Prn1 is absent, and not detected in the treated SN250 strain, in which Prn1 is highly increased, while Nrg1, which is an Mnl1 antagonist, was only detected in the treated SN250 strain (Figure S4). In the *prn1∆* strain, Mnl1 was detected only under oxidative stress, together with the increase in three Mnl1-regulated oxidative stress response proteins. In agreement with the absence of detection of Nrg1 in the *prn1∆* strain, 35 Nrg1-repressed proteins presented a significant increase in abundance (Figure S4). Among these thirty-five proteins, there were six proteins with oxidoreductase activity (Ali1, Gpd1, Gre2, Oye23, Sod1, and Yah1). Moreover, Nrg1 was only detected in the SN250 strain, and nine Nrg1 down-regulated proteins were detected only in the SN250 strain under H_2_O_2_ treatment. Detailed lists of proteins regulated either by Mnl1 or Nrg1 identified under each condition with their relative abundances are provided in Table S8.

In mammals, Pirin exerts its function in the oxidative stress response through Nrf2 modulation [18], but a *C. albicans* Nrf2 orthologue has not yet been described. Further experiments will determine whether Mnl1 could be the Nrf2 homologue.

### 4.5. Prn1 May Be Related to Mitochondrial Oxidative Stress Detoxication in Basal Conditions

Comparative analysis of the *prn1∆* mutant proteome with respect to the SN250 strain in the absence of oxidative stress revealed that mitochondrial proteins with significant changes in abundance in the *prn1∆* strain were related to the mitochondrial oxidative stress response (e.g., chaperones) and mitochondrial ribosome, while in the SN250 strain, these proteins were involved in mitochondrial cell redox regulation and mitochondrial respiratory function (Supplemental Figure S3A). These results may indicate that in basal conditions, although Prn1 absence does not promote dysfunction in mitochondrial membrane polarization, it might be involved in mitochondrial ROS detoxication produced by the electron transport chain function. In *Streptomyces*, PirA has been identified as a negative regulator of the beta-oxidation pathway, decreasing oxidative stress [21]. In eukaryotes, this process is produced in the inner mitochondrial membrane, so Prn1 could have the same function in *C. albicans*, regulation of the oxidative stress. Future studies will focus on determining the mitochondrial Prn1 function in the absence of oxidative stress.

Mnl1 and orf19.1150 transcription factors also presented a significant difference in abundance between the two strains in the absence of H_2_O_2_. Another transcription factor, Swi3, a subunit of the SWI/SNF chromatin remodeling complex required for the expression of different genes in *S. cerevisiae* [40], significantly increased in abundance in the SN250 strain.

### 4.6. Involvement in the Oxidative Stress Response of orf19.4850, an S. cerevisiae CUB1 Orthologue

In-depth analysis of proteins expressed in the SN250 strain after treatment (Table 2) highlighted *S. cerevisiae CUB1* orthologue orf19.4850, which was exclusively detected in this strain and this condition. In *S. cerevisiae*, this protein is involved in DNA repair and proteasome function [26]. However, the molecular function, biological process, and molecular component of orf19.4850 remain unknown for *C. albicans*. Our experiments support a possible role of this protein in the oxidative stress response and conserved proteasome-related function (Figure 7). The fact that it was detected exclusively when Prn1 increased its abundance suggests a possible relationship between both proteins in the oxidative stress response.

## 5. Conclusions

Taken together, these results reveal that Prn1 is a relevant component of the oxidative stress response in *C. albicans*. The function of Prn1 under basal conditions seems to be related to the regulation of mitochondrial redox. After H_2_O_2_ treatment, this protein significantly increases in abundance, correlating with the need for ROS detoxification, which leads to an improvement in cell survival and recovery after stress. Under oxidative stress in the absence of Prn1, proteasome activity is increased, translation-related proteins decrease in abundance, and several transcription factors change in abundance with respect to the wild-type strain. In addition, we postulate a possible relationship between Prn1 and the *C. albicans* protein orthologue to *S. cerevisiae*, Cub1.

## Figures and Tables

**Table 1:** Top 10 proteins with the greatest increase in relative abundance in after treatment in the SN250 or *prn1∆* strain compared with the control condition and the other strain.

Strain	Protein	Description	SN250 H_2_O_2_/ SN250 Control Ratio Log_2_	*prn1∆* H_2_O_2_/ *prn1∆* Control Ratio Log_2_
SN250	Psa2	Mannose-1-phosphate guanyltransferase	5.36	NS
	Icl1	Isocitrate lyase; glyoxylate cycle enzyme	4.63	3.82
	Prn1	Protein with similarity to Pirin	4.16	ND
	Alt1	Putative alanine transaminase	4.13	NS
	Oye22	Putative NADPH dehydrogenase	4.06	3.28
	Acc1	Putative acetyl-coenzyme-A carboxylases	4.04	3.57
	orf19.3477	Putative pseudouridine synthase	4	3.54
SN250 and *prn1∆*	Cip1	Possible oxidoreductase	4.47	4.65
	Ahc1	Ortholog(s) histone acetyltransferase activity	4.13	4.23
	Pim1	ATP-dependent Lon protease	3.96	4.17
*prn1∆*	Mal2	Alpha-glucosidase	4.57	+
	Lys21	Homocitrate synthase	4.34	3.07
	orf19.6035	Unknown function	4.27	3.89
	Hsp21	Small heat shock protein	4.25	3.19
	Qcr9	Putative ubiquinol cytochrome c reductase	4.19	NS
	Nuc2	Putative NADH-ubiquinone oxidoreductase	4.15	+
	orf19.7310	Role in directing meiotic recombination	4.09	NS

“+” indicates proteins detected only in the treated condition, “NS” indicates non-significant change in abundance, and “ND” indicates non-detected proteins. Prn1 ratio abundances have been bolded with grey background.

**Table 2:** Proteins detected in one condition but not in the other and without significant change in abundance or not detected in the other strain.

Strain	Detected in Treated Condition	Detected in Basal Condition
SN250 H_2_O_2_/ SN250 control	Ald4, Bzz1, orf19.4430, orf19.4850, Rib2	Hrr25, Mak11, Mcm3, orf19.1272, Ynd1
*prn1∆* H_2_O_2_/ *prn1∆* control	Fmp28, Nit2, orf19.1150, orf19.2051, orf19.2452, Osh2, Phb2, Pr26, Rpn3, Rpt4, Rts1, Smp2, Snu66, Tad2, Tif33, Uso1, Yah1	Afg2, Has1, Lig1, Msd1, Nrg1, orf19.5987, orf19.6445, Pnc1, Rpa43, Sec24, Trp1, Ubp12

Protein descriptions and quantification of relative abundance are found in Table S5.

**Table 3:** Proteins that presented opposite changes in abundance between both strains after H_2_O_2_ treatment.

Protein	Description	SN250 H_2_O_2_/ SN250 Control Ratio Log_2_	*prn1∆* H_2_O_2_/ *prn1∆* Control Ratio Log_2_
Bas1	Putative helix–loop–helix (HLH) transcription factor; role in filamentous growth	-	3.61
Cat5	2-octoprenyl-3-methyl-6-methoxy-1,4-benzoquinone hydroxylase activity; ubiquinone biosynthetic process	-	2.75
Dad4	Subunit of the Dam1 (DASH) complex; chromosome segregation	−3.15	4.06
Fmp53	Ubiquinone-6 biosynthetic process	3.69	-
Ftr2	High-affinity iron permease	−2.67	2.11
Mfg1	Regulator of filamentous growth; biofilm formation	-	3.5
Mnl1	Transcription factor; induces transcripts of stress response genes via SLE (STRE-like) elements	-	+
orf19.764	Ortholog(s)-negative regulation of TORC1 signaling	-	+
Rpo41	Putative mitochondrial RNA polymerase; repressed in core stress response	-	3.45

“+” indicates proteins only detected in the treated condition; “-” indicates proteins only detected in the control condition.

## Data Availability

The dataset from the proteomics analysis has been deposited in the ProteomeXchange Consortium via the PRIDE partner repository with the dataset identifier PXD040804. A previous pre-print version of this manuscript was deposited on 8 November 2023, at BioRxiv project DOI: https://doi.org/10.1101/2023.11.07.566035.

## Supplementary Materials

The following supporting information can be downloaded at https://www.mdpi.com/article/10.3390/antiox13050527/s1: Table S1: List of 1806 quantified proteins between SN250 and *prn1D* strains with Proteome Discoverer software using the *Candida* Genome Database; Table S2: List of proteins that significantly changed their abundance q value < 0.05 in response to the treatment in SN250 and *prn1∆* strain; Table S3: List of proteins that significantly changed their abundance q value < 0.05 only in SN250 or *prn1∆* strain after treatment; Figure S1: Heat map containing decreased nucleotide metabolism GO Term proteins after 200 min under 10 mM H_2_O_2_ treatment in each strain; Figure S2: Heat maps containing (a) increased oxidoreductase activity proteins in SN250 strain (b) increased protein catabolic process proteins in *prn1D* strain after 200 min under 10 mM H_2_O_2_ treatment; Table S4: List of oxidoreductase activity proteins that significantly increased their abundance q value < 0.05 in SN250 and in *prn1∆* strain after treatment; Table S5: List of proteins detected in one condition but not even detected on the other one for each strain; Figure S3: (A) Heat maps containing proteins that significantly changed their abundance between control strains grouped by Gene Ontology Terms (B) Mitochondrial membrane depolarization using JC-1 dye measured by flow cytometry in SN250 and *prn1D* untreated strains and after 15, 25 or 50 min under 10 mM H_2_O_2_ treatment. The graph presents the most representative of three biological replicates; Table S6: List of proteins that significantly changed their abundance q value < 0.05 between both strains in the absence or the presence of the oxidative stress; Figure S4: List of proteins red by Mnl1 and Nrg1 increased in *prn1D* H_2_O_2_ respect to (a) *prn1D* control and (b) SN250 H_2_O_2_. The relative ratio for each protein is presented in Table S1; Figure S5: Human pirin and *C. albicans* Prn1 alignment using BLAST. Prn1 presents a partial homology with human pirins (62% positives residues). Table S7: Yeast retrograde response pathway proteins; Table S8: List of significantly increased proteins in *prn1D* H_2_O_2_ regulated by Mnl1 and Nrg1 transcription factors.
